# Individual stress reactivity predicts alcohol craving and alcohol consumption in alcohol use disorder in experimental and real-life settings

**DOI:** 10.1038/s41398-025-03447-8

**Published:** 2025-07-03

**Authors:** Judith Zaiser, Sabine Hoffmann, Sina Zimmermann, Tatjana Gessner, Milena Deck, Nina Kim Bekier, Martin Abel, Philipp Radler, Jens Langejürgen, Bernd Lenz, Sabine Vollstädt-Klein, Jan Stallkamp, Clemens Kirschbaum, Falk Kiefer, Patrick Bach

**Affiliations:** 1 Department of Addictive Behavior and Addiction Medicine, Central Institute of Mental Health, Medical Faculty Mannheim / Heidelberg University, Heidelberg, Germany; 2 German Center for Mental Health (DZPG), partner site Heidelberg/Ulm, Heidelberg, Germany; 3 Department of Biostatistics, Central Institute of Mental Health, Medical Faculty Mannheim / Heidelberg University, Heidelberg, Germany; 4 Fraunhofer Institute for Manufacturing Engineering and Automation IPA, Mannheim, Germany; 5 Feuerlein Center on Translational Addiction Medicine (FCTS), University of Heidelberg, Heidelberg, Germany; 6 Mannheim Institute for Intelligent Systems in Medicine, Medical Faculty Mannheim, Heidelberg University, Heidelberg, Germany; 7 Department of Psychology, Technical University Dresden, Dresden, Germany

**Keywords:** Addiction, Prognostic markers

## Abstract

Stress- and alcohol cues trigger alcohol craving and alcohol consumption in alcohol use disorder (AUD). However, their interactions on a physiological and psychological level and their effects on daily alcohol craving and alcohol use in real-life situations are not understood yet. We conducted a randomized-controlled experimental study to compare the effects of psychosocial stress against physical stress and a control intervention, each followed by an alcohol cue-exposure, on alcohol craving, subjective stress and saliva cortisol levels (main outcomes) in *N* = 121 individuals with AUD and collected data on daily alcohol use and craving during a 1-year ambulatory assessment phase. We applied linear mixed models to compare the effects of experimental interventions on the main outcomes and the relative contributions of the observed changes on the main outcomes to predicting stress and alcohol craving during the experiment and alcohol use and craving during the ambulatory assessment phase. Sequential exposure to psychosocial stress and alcohol cues induced higher cortisol levels (F_(10,580)_ = 10.819, *p* < 0.001), subjective stress (F_(2,117)_ = 10.520, *p* < 0.001) and alcohol craving (F_(6,348)_ = 4.313, *p* < 0.001) compared to the exposure to physical stress and the control condition. Subjective stress reactivity was the most influential predictor of craving during the experiment (F_(1,92)_ = 9.43, *p* = 0.003) and during the ambulatory phase (β = 0.16, *p* = 0.039) while cortisol levels predicted alcohol consumption in real-life settings (β = 9.76, *p* = 0.043). Our results highlight the impact of psychosocial stress on cue-induced craving and subjective and neuroendocrine stress responses and demonstrate links between subjective and neuroendocrine stress-reactivity and alcohol craving and alcohol use in real-life settings.

## Introduction

Alcohol Use Disorder (AUD) is a chronic and relapsing disease in which stress- and alcohol cue-exposure have been identified as triggers for alcohol craving and subsequent alcohol use [1–5]. Regarding the effects of stress in AUD, it has been shown that exposure to acute psychosocial stress increases alcohol craving and alcohol consumption [6, 7]. Specifically, exposure to the Trier Social Stress Test (TSST), an established experimental model for the induction of psychosocial stress [8], was found to increase alcohol use in social drinkers [9] and alcohol craving in heavy drinkers with AUD [10]. On a neuroendocrine level, the exposure to psychosocial stress was found to induce increased hypothalamus-pituitary-adrenal (HPA) axis activity and cortisol release, as well as higher sympathetic-adrenal-medullary activity [11, 12] and higher subjective stress levels (see [13] for review). It has been shown that experimental exposure to visual alcohol cues increases alcohol craving and the motivation to drink alcohol [14] in treatment seeking individuals with AUD. On a physiological level, alcohol cue exposure was found to induce higher activation in the mesolimbic reward system, which showed close associations with the subjective experience of alcohol craving [15, 16]. Beyond the effects of stress and alcohol cues alone, studies indicated significant interactions between both in AUD. In this context, it was shown that sequential exposure to psychosocial stress (i.e. the TSST) and alcohol cues induces higher neuroendocrine stress responses (i.e. cortisol release), compared to the exposure to either stress or alcohol cue in individuals with AUD and comorbid post-traumatic stress disorder. This indicates differential responsiveness of the neuroendocrine stress system to combined stress- and alcohol cue-exposure [17]. In the same study, sequential stress- and alcohol cue-exposure and imagery of stressful situations or guided alcohol imagery all induced higher craving for alcohol, compared to a guided neutral imagery, supporting the impact of stress and alcohol cue-exposure on cue-induced craving. Regarding the interaction between the effects of stress and drug cue-exposure in substance use disorders in general, a recent study in cocaine use disorder showed that exposure to psychosocial stress enhanced cocaine cue-induced craving, pointing towards additive effects of stress and substance cue exposure on substance craving [18].

Taken together, previous studies indicated that exposure to stress and alcohol cues trigger alcohol use and alcohol craving and that both interact to create distinct neuroendocrine and subjective stress reactions. However, the understanding of the mechanisms underlying the interaction between stress- and alcohol cue-exposure and their impact on everyday alcohol craving and alcohol use are not understood yet. To address this question, we investigated stress- and alcohol cue-induced effects on physiological and psychological stress responses in a randomized controlled experimental study and determined their impact on alcohol craving and alcohol use in everyday-life during a one-year ambulatory assessment phase. We hypothesized that psychosocial stress, combined with alcohol cue-exposure, induces higher cortisol reactivity compared to physical stress and a matched control condition, combined with alcohol cue-exposure, and that subjective and neuroendocrine (i.e. cortisol) stress reactivity predict alcohol craving and alcohol consumption in real-life settings during the one-year ambulatory follow-up.

## Methods and materials

### Study sample

A total of 121 participants were recruited from a multi-center cohort study between May 2020 and September 2022 in the framework of a Collaborative Research Centre on ‘Loosing and Regaining Control over Drug Intake’ (TRR265; sites: Charité-Universitätsmedizin Berlin, Technical University Dresden, and Central Institute of Mental Health (CIMH) Mannheim). Participants of the cohort study were recruited from the general population. The cohort study consisted of four study visits (baseline and three follow-up visits) and real-life ecological momentary assessments (EMA) for one consecutive year [19]. The current sub-study introduced an additional visit between baseline and first follow-up visit. In order to be considered for participation in the current study, a mild to severe AUD, according to the Diagnostic and Statistical Manual of Mental Disorders (DSM-5) [20], was required, in which the majority (81%) fulfilled criteria for mild to moderate AUD which according to DSM-IV terminology would translate to alcohol abuse and alcohol dependence (for further eligibility criteria see Supplements). For the present sub-study, we aimed to enroll about every second participant of the cohort sample from the Mannheim study site (*N* = 276) (i.e. half of the cohort were randomly selected and re-contacted). Of *N* = 138 contacted participants, we successfully enrolled *N* = 121 in the here described sub-study (see Supplementary Figures, Figure S3 for CONSORT study flow chart). Both studies were purely observational thus no specific intervention or treatment was applied and no reduction goals were assigned.

### Study design

**Fig. 1:**
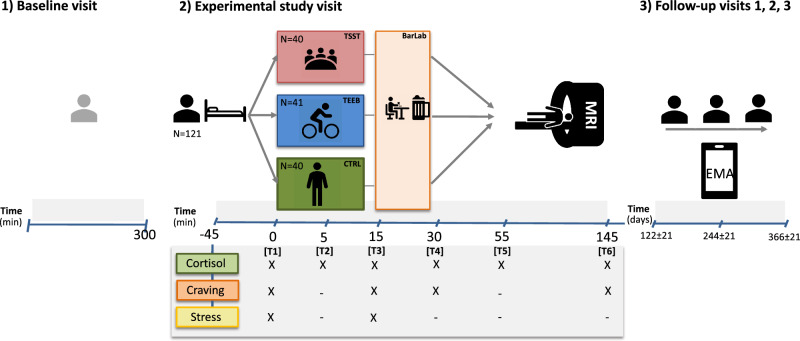
Study design. 1 A baseline visit (*N* = 267) preceded the here presented study and is included in this depiction for completeness. 2 *N* = 121 participants were enrolled in this randomized-controlled longitudinal study. The study commenced with a rest period, followed by the randomization to one of three stress conditions in which participants were exposed to psychosocial stress (TSST), a matching control condition (CTRL) or to physical stress (training on an endurance exercise bike (TEEB)). Subsequently, alcohol cue exposure in a laboratory designed to look like a bar (BarLab) and a fMRI scanning was conducted. 3 For the following year, participants were prompted on a regular basis to insert data regarding drinking behavior via EMA.

A 1:1:1 randomized-controlled experimental study design with three parallel groups was applied. The study consisted of four main phases: A rest period (preceding T1), followed by stress exposure respectively control condition (T1) and alcohol cue exposure in a laboratory bar setting (T3). Subsequently, a fMRI session including a cue-exposure paradigm was conducted which is reported elsewhere [21]. Real-life alcohol use and alcohol craving were assessed during a one-year follow-up every other day using ecological momentary assessments (see Fig. 1). The aim of the study was to measure the effects of psychosocial stress and alcohol cue exposure on subjective stress, alcohol craving, and neuroendocrine response. Furthermore, this study attempted to associate experimental findings to ecological data of alcohol consumption and craving.

The study was approved by Ethics Committee of the University of Heidelberg (approval No. 2018-626N-MA) and preregistered at clinical trials (NCT03810924). All participants provided written informed consent according to the Declaration of Helsinki and all methods were performed in accordance with the relevant guidelines and regulations.

### Experimental procedure

Participants were randomized to one of three experimental groups using a block-wise randomization procedure: i) TSST [8] (*N* = 40), ii) a matched control condition (CTRL) (*N* = 40), iii) physical stress, induced by training with an endurance exercise bike (TEEB) (*N* = 41). The TSST is an established paradigm for psychosocial stress exposure in a laboratory that is defined as uncontrollable, unpredictable and a threat to self-esteem [8, 22, 23]. We opted use the TSST and combine it with an alcohol cue-exposure, because of higher effect on stress responses (especially neuroendocrine), compared to other stressors (e.g. guided) [17]. A detailed description of the procedure can be found elsewhere [8]. The control condition consisted of a similar procedure [24] without the evaluative feedback which is expected to be the main factor of psychosocial stress induction [8]. The TEEB condition represented an active control condition aiming to disentangle the similar subjective and neuroendocrine responses which were found in prior studies as responses to psychosocial and physiological stressors while effects on alcohol consumption in AUD were different [25–28]. The TEEB condition included a warm up phase (2,5 min) followed by a ride (8 min) with 60%–75% of the individual maximum heart rate [29, 30] and aimed to expose participants to a stressor which yield similar physiological arousal levels as TSST but without the social evaluative component. Directly after that, participants were transferred to a bar lab (i.e. a room resembling a bar) and were exposed to their favorite drink for 3 min following the procedure established previously [17, 31]. More detailed description of the experimental procedure can be found in the supplement section.

### Main outcome measures – experimental study

Alcohol craving (measured via Alcohol Urge Questionnaire (AUQ) [32]), subjective stress (measured via Primary Appraisal Secondary Appraisal (PASA) [33]) and cortisol levels (saliva cortisol [34]) served as the main outcome measures during the experiment. All parameters were assessed at T1 to obtain a baseline level and also repeatedly throughout the experiment (see Fig. 1). For details of preanalytical measures and analysis see Supplementary Methods. Saliva cortisol data showed a skewed distribution. Therefore the data were processed via logarithmic transformation (log) for statistical analysis [35, 36].

### Main outcome measures – ambulatory assessment phase

Data on daily craving and alcohol consumption were collected over a duration of one year (see Fig. 1) via EMA. Participants were asked to report their drinking behavior (what was consumed, how much) every other day referring to the last two days. The compliance rate of the collected data was 78% over the one-year observational period after the experiment (determined by the proportion of answered prompts compared to the total amount of prompts). For more information on EMA assessment, see supplements.

### Statistical analysis

#### Sample size estimation

We were able to enroll *N* = 121 of the planned *N* = 150 participants. The sub-perfect enrollment resulted from Covid-19 associated restrictions, which caused a later-than-expected start of the recruitment. Still, post-hoc power analyses, considering a repeated measures analyses of variance model used for the primary analyses, indicated that with a sample of *N* = 121 (with *n* > = 40 per group) and > = 2 repeated assessments of the outcome, the analyses still yield a power of >90% for detecting between-within interactions with at least medium effect size (f > = 0.2), which were considered already in the initial a-priori sample size estimation, based on prior evidence [17].

#### Analyses of stress- and cue-induced effects on craving, subjective and neuroendocrine stress responses during the experiment

The primary analyses investigated the between-within interaction of experimental group and time points (see Fig. 1) on the main outcome measures (AUQ, PASA, cortisol) using linear mixed models, as implemented in the Statistical Package for the Social Sciences software (SPSS, IBM Corp., Somers, NY, USA) version 29.0 (SPSS 29.0). Linear mixed models were fitted for all three main outcome variables (AUQ with 4 assessments, PASA with 2 assessments and cortisol with 6 assessments) as dependent variables with group and time points as predictors, including a random intercept. No additional covariates were considered in the primary analyses, as randomized group assignment yielded comparable groups. Still, we also conducted additional sensitivity analyses considering age and sex as covariates. We applied the restricted maximum likelihood (REML) method to estimate the fixed effect parameters and the Kenward-Roger approximation to estimate the denominator degrees of freedom. We report F-tests for the fixed effects in the linear mixed models and the corresponding parameter estimates β and conducted Bonferroni-corrected post hoc comparison between groups and time points.

#### Analyses of predictors of stress- and craving-reactivity during the experiment

Associations between the main outcome measures were assessed using linear mixed models in SPSS. To this end, linear mixed models were fitted separately for each of the main outcome measures (AUQ, PASA, cortisol) as dependent variable with the corresponding other main outcomes, gender, group, age, number of AUD criteria, sub-chronic stress (PSS), starting time of the experimental procedure and blood pressure as predictors, including a random intercept. We assessed an additional model including the starting time of the experiment since previous research identified the association of cortisol to the circadian rhythm with fluctuating hormonal release over the day [37].

#### Analyses of predictors of alcohol use and alcohol craving during the one-year ambulatory assessment phase

Associations between stress- and cue-reactivity during the experiment with alcohol craving and consumption in real-life settings were assessed using linear mixed models in SPSS.

Linear mixed models were fitted for daily alcohol craving (i = 11257) and daily alcohol consumption (i = 22653) during the one-year ambulatory phase as dependent variables in two separate models with average AUQ scores, average PASA scores and average cortisol levels across the experimental stress- and alcohol cue-exposure, as well as gender, group, age, weekend days versus weekdays, number of AUD criteria, sub-chronic stress (PSS) and blood pressure as predictors, including a random intercept. We added a categorical predictor to model the effects of weekdays versus weekends on the dependent variable, in order to control for the observed significant weekend-weekday pattern of alcohol consumption in overall cohort sample [38].

#### Analyses of sample characteristics

We characterized the given sample by applying univariate analyses of variance and crosstabs with Fisher’s exact Test. Pearson correlation analyses were conducted to identify associations between the main outcome measures for acute craving and situational stress. All analyses were conducted using SPSS version 29.0.

## Results

### Sample characteristics and substance use patterns

We assessed the associations between parameters regarding demographics, alcohol use and severity measures for *N* = 121 participants; we did not observe any significant differences between the three experimental groups at baseline (see Table 1). The here described subsample was randomly drawn (i.e. approximately every second participant) from the cohort sample (*N* = 276) recruited at the Mannheim study site and did not differ from the overall cohort-sample on any of the investigated variables (see Supplementary Tables, Table S1).

**Table 1:** Demographic data, alcohol use, and severity measures for all three groups.

Subgroup	1	2	3		
	TSST (*n* = 40)	TEEB (*n* = 41)	Control (*n* = 40)	Statistics	Significance
*Demographical variables*					
Gender (female; male)	13; 27	16; 25	16; 24	Z = 0.60	*p* = 0.794
Age (years)	38.08 (12.50)	38.61 (13.84)	38.62 (13.73)	*F* (2, 120) = 0.02	*p* = 0.978
Education (left school without a diploma /currently attending school/attended higher education)	0/2/36	0/1/34	0/1/38	Z = 0.66	*p* = 0.841
*Substance use patterns*					
AUD criteria last 12 months	3.78 (1.67)	4.16 (1.50)	4.10 (1.54)	*F* (2, 116) = 0.68	*p* = 0.507
AUDIT	14.11 (5.42)	14.97 (6.29)	16.87 (5.11)	*F* (2, 103) = 2.37	*p* = 0.099
Alcohol consumption - last 3 months [g alcohol/day]	6.05 (3.49)	5.85 (3.43)	6.71 (3.52)	*F* (2, 116) = 0.65	*p* = 0.527
Alcohol consumption - typical weekday [g alcohol/day]	3.88 (2.72)	3.14 (2.17)	3.72 (2.85)	*F* (2, 116) = 0.87	*p* = 0.423
Alcohol consumption - typical weekend [g alcohol/day]	7.36 (4.31)	7.30 (3.83)	7.30 (3.89)	*F* (2, 116) = 0.00	*p* = 0.997
Smoker (yes/no)	11; 25	11; 19	13; 26	Z = 0.32	*p* = 0.811
FTND	0.91 (1.58)	2.36 (2.66)	1.69 (2.06)	*F* (2, 34) = 1.28	*p* = 0.293
Cannabis use lifetime [yes; no]	31; 9	29; 9	27; 12	Z = 0.83	*p* = 0.721
Cannabis use last 3 months [yes; no]	7; 24	11; 18	9; 18	Z = 5.40	*p* = 0.761
Cannabis drug test [pos.; unknown; neg.]	0; 0; 40	2; 1; 38	1; 1; 38	Z = 3.11	*p* = 0.667
*Clinical scales*					
PSS	16.38 (6.79)	17.10 (6.78)	14.97 (8.02)	*F* (2, 103) = 0.76	*p* = 0.470
ADS	7.50 (3.94)	9.27 (5.58)	8.43 (4.18)	*F* (2, 120) = 1.48	*p* = 0.233
OCDS	6.65 (3.65)	8.46 (5.05)	8.55 (4.13)	*F* (2, 120) = 2.47	*p* = 0.089
BSI	3.42 (3.74)	3.60 (4.12)	3.32 (3.55)	*F* (2, 103) = 0.08	*p* = *0*.925
STAI Trait	39.61 (11.42)	39.46 (11.00)	38.08 (10.58)	*F* (2, 111) = 0.23	*p* = 0.799
CTS	7.70 (3.23)	7.37 (2.74)	7.87 (3.70)	*F* (2, 116) = 0.24	*p* = 0.788
*Experimental Session - Baseline Values (Differences)*					
AUQ	12.80 (4.50)	14.35 (6.70)	13.98 (7.50)	*F* (2, 119) = 0.65	*p* = 0.526
PASA	−2.06 (1.05)	−1.85 (1.42)	−2.06 (1.21)	*F* (2, 119) = 0.37	*p* = 0.691
Cortisol (log)	4.01 (2.43)	4.15 (2.74)	4.42 (2.71)	*F* (2, 119) = 0.24	*p* = 0.787
*EMA*					
Compliance (in %)	84,6%	82,7%	76,7%	*F* (2,551.05) = 1.92	*p* = 0.152

Gender: None of the participants assigned themselves to “divers”.

*ADS* alcohol dependence scale, *BSI* brief symptom inventory, *FTND* Fagerstroem test for nicotine Dependence, *OCDS* obsessive-compulsive drinking scale, *STAI* state-trait-anxiety inventory, *AUQ* alcohol urge questionnaire, *PASA* primary appraisal secondary appraisal, *CTS* Childhood Trauma Screener, *EMA* ecological momentary assessment, ‘higher education’ means education following high school, *SD* standard deviation.

### The effects of combined stress- and cue-exposure on alcohol craving, subjective stress experience and neuroendocrine stress response

**Fig. 2:**
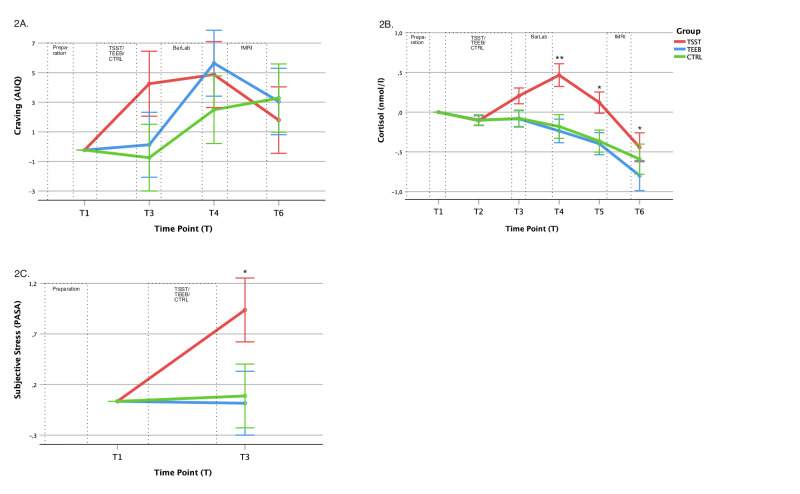
The interaction of time and group for the main outcomes. Depiction of the interaction between time and group on **A** alcohol craving (F(6,348.583) = 4.313, p < 0.001; error bars: 95% CI), **B** cortisol levels (log cortisol; (F(10,580.420) = 10.819, p < 0.001; error bars: 95% CI), and **C** subjective stress levels (F(2,117.197) = 10.520, p < 0.001; error bars: 95% CI).

The interaction of *group* and *time* was significant for all main outcomes during the experiment with different magnitudes (see Table 2 and Fig. 2A–C): Acute craving (AUQ) (F_(6,348)_ = 4.313, p < 0.001), saliva cortisol (F_(10,580)_ = 10.819, p < 0.001), subjective stress (PASA) (F_(2,117)_ = 10.520, p < 0.001). We also found a significant effect for systolic blood pressure (F_(8, 462)_ = 2.421, p = 0.014) (see Supplementary Tables, Table S2). Sensitivity analyses considering age and sex as covariates confirmed the significance of the observed interaction effects (not reported).

**Table 2:** Multilevel Modelling Results for the effect of experimental intervention on craving, cortisol release and subjective stress experience.

	F (df1, df2)	p
**Craving (AUQ)**		
Intercept	592.89 (1, 118.13)	**<0.001**
Group	0.39 (2, 118.13)	0.681
Timepoint (T)	20.26 (3, 348.59)	**<0.001**
Group* T	4.31 (6, 348.58)	**<0.001**
**Cortisol (log)**		
Intercept	593.83 (1, 118.13)	**<0.001**
Group	3.36 (2, 118.13)	**0.038**
Timepoint (T)	75.49 (5, 580.42)	**<0.001**
Group* T	10.82 (10, 580.42)	**<0.001**
**Subjective Stress (PASA)**		
Intercept	268.04 (1, 117.92)	**<0.001**
Group	1.23 (2, 117.92)	0.297
Timepoint (T)	11.32 (1, 117.2)	**<0.001**
Group* T	10.52 (2, 117.2)	**<0.001**

Repeated assessments (see Fig. 1): AUQ *n* = 4, cortisol (log) *n* = 6, PASA *n* = 2; 95% CI, 95% confidence interval. p < 0.05 in bold.

*AUQ* alcohol urge questionnaire, *Group* stress condition, *PASA* primary appraisal secondary appraisal.

Post-hoc analyses comparing the three experimental groups at single time points (T1-T6) demonstrated that directly after the stress challenge (T3), psychosocial stress compared to physical stress and the control condition induced higher subjective stress levels (F: 4.85, p_TSST vs. TEEB_ = 0.037; p_TSST vs CTRL_ = 0.015). After the following alcohol cue-exposure (T4), we observed significantly higher saliva cortisol levels in the psychosocial stress group compared to the other two groups (F: 15.64, p_TSST vs. TEEB_ < 0.001; p_TSST vs CTRL_ < 0.001). Cortisol levels in the psychosocial stress group were significantly higher compared to the other two groups until the end of the experimental session (T5: F = 8.64, p_TSST vs. TEEB_ = 0.001; p_TSST vs. CTRL_ = 0.002; T6: F = 3.06, p_TSST vs. TEEB_ = 0.048). After Bonferroni correction, effect for craving did not remain significant.

### Predictors of craving- and stress-reactivity during the experiment

Increased subjective stress experience predicted alcohol craving (AUQ) (β = 1.91, p = 0.003). Craving increased over the time of the experiment (T3: β = 1.7, p = 0.033; T4: β = 4.82, p < 0.001) independent of group. Male gender (β = 0.27, p = 0.011) predicted cortisol release. Controlling for the starting time of the experimental procedure, in addition to male gender also the starting time (β = −2,482e^-5^; p < 0.001), psychosocial stress (β = 0.28, p = 0.012) and the number of met AUD-criteria (β = 0.07, p = 0.019) predicted cortisol release (see Supplementary Tables, Table S3).

Craving (AUQ) (β = 0.04, p < 0.001) and prior stress scores (PSS) (β = 0.07, p < 0.001) predicted subjective stress (PASA) which increased over time (β = 0.26, p = 0.042) Table 3.

**Table 3:** Multilevel Modelling Results for identifying predictors of craving and stress.

	β [95% CI]	p	F (df1, df2)	p
**Craving (AUQ)**				
Intercept	10.82 [1.81; 19.84]	**0.019**	9.89 (1; 161.67)	**0.002**
Gender			1.22 (1; 96.96)	0.273
Male	1.55 [−1.24; 4.35]			
Female	[Reference]			
Group			0.43 (2;92.61)	0.654
TSST	0.65 [−2.57; 3.87]	0.689		
TEEB	1.49 [−1.72; 4.71]	0.358		
CTRL	[Reference]			
Age	−0.1 [−0.2; 0.01]		3.24 (1;101.13)	0.075
AUD criteria	0.51 [−0.37; 1.38]		1.33 (1;92.94)	0.252
PSS	0.12 [−0.07; 0.31]		1.67 (1;92.21)	0.200
Timepoint (T)			22.15 (2;213.41)	**<0.001**
T1	[Reference]			
T3	1.7 [0.14; 3.27]	0.**033**		
T4	4.82 [3.35; 6.29]	**<0.001**		
BP (sys)	0.13 [−0.05; 0.07]		0.2 (1;247.95)	0.657
Cortisol (log)	−1.62 [−3.33; 0.1]		3.45 (1;282.13)	0.064
PASA	1.91 [0.68; 3.15]		9.43 (1;92.45)	**0.003**
**Cortisol (log)**				
Intercept	1.11 [0.47; 1.75]	**<0.001**	17.59 (1;167.54)	**<0.001**
Gender			6.69 (1;94.11)	**0.011**
Male	0.27 [0.06; 0.47]			
Female	[Reference]			
Group			2.25 (2;91.3)	0.111
TSST	0.22 [−0.02; 0.46]	0.069		
TEEB	−0.02 [−0.26; 0.22]	0.882		
CTRL	[Reference]			
Age	−0.01 [−0.02; 0.00]		3.56 (1;99.49)	0.062
AUD criteria	0.05 [−0.01; 0.12]		2.75 (1;91.69)	0.101
PSS	0.01 [0;0.03]		2.29 (1;91.28)	0.134
Timepoint (T)			1.72 (2;218.4)	0.182
T1	[Reference]			
T3	0.04 [−0.06; 0.14]	0.433		
T4	0.09 [−0.01; 0.2]	0.068		
BP (sys)	0 [−0.01; 0]		0.12 (1; 273.74)	0.729
AUQ	−0.01 [−0.01; 0]		2.28 (1;287.68)	0.132
PASA	0.02 [−0.07; 0.11]		0.17 (1;95.3)	0.685
**Subjective Stress (PASA)**				
Intercept	−3.88 [−5.41; −2.35]	**<0.001**	23.98 (1;147.68)	**<0.001**
Gender			3.49 (1;100.2)	0.065
Male	−0.43 [−0.89; 0.03]			
Female	[Reference]			
Group			0.283 (2;94.67)	0.754
TSST	0.19 [−0.31; 0.68]	0.454		
TEEB	0.08 [−0.43; 0.6]	0.750		
CTRL	[Reference]			
Age	0 [−0.02; 0.02]		0 (1;103.09)	0.956
AUD criteria	−0.11 [−0.25; 0.03]		2.48 (1;96.22)	0.119
PSS	0.07 [0.04; 0.1]		18.71 (1;97.73)	**<0.001**
Timepoint (T)			4.2 (1;130.05)	**0.042**
T1	[Reference]			
T3	0.26 [0.01; 0.5]	**0.042**		
BP (sys)	0 [−0.01; 0.01]		0.39 (1;176.52)	0.533
AUQ	0.04 [0.02; 0.06]		11.3 (1;187.17)	**<0.001**
Cortisol (log)	0.31 [−0.03; 0.65]		3.17 (1;173.66)	0.077

95% CI, 95% confidence interval. p < 0.05 in bold.

*AUQ* alcohol urge questionnaire, *group* stress condition, *TSST* trier social stress test, *TEEB* training endurance exercise bike, *CTRL* control, *AUD* alcohol use disorder, *PSS* perceived stress scale, *BP (sys)* systolic blood pressure, *PASA* primary appraisal secondary appraisal.

### Predictors of alcohol use and alcohol craving during the one-year ambulatory assessment phase

Alcohol craving (AUQ) during the stress experiment predicted alcohol craving during follow-up (i = 11257; EMA data) (β = 0.05, 95% CI 0.02-0.07; p < 0.001). Higher age (β = 0.02, 95% CI 0-0.03; p = 0.014), increased subjective stress experiences (β = 0.16, 95% CI 0.01-0.32; p = 0.039) and weekend days (β = 0.1, 95% CI 0.07-0.14; p < 0.001) also showed a positive association with alcohol craving (listed results can be found at Supplementary Tables, Table S4 and Supplementary Figures, Figure S1).

Elevated mean cortisol during the experimental session (β = 9.76, 95% CI 0.3–19.23; p = 0.043), higher age (β = 0.39, 95% CI 0.02–0.77; p = 0.038) and systolic blood pressure (β = 0.32, 95% CI 0.04–0.61; p = 0.027) predicted daily alcohol consumption (i = 22653). This association remained significant, when controlling for weekend versus weekdays in the model, which showed a significant association with alcohol consumption (reference weekdays: β = 14.03, 95% CI 12.98–15.09; p < 0.001) (listed results can be found at Supplementary Tables, Table S4 and Supplementary Figures, Figure S2).

## Discussion

Results of this randomized-controlled experimental study highlight the interaction effects of stress- and alcohol cue-exposure on neuroendocrine and subjective stress-reactivity and alcohol craving. In addition, results provide compelling evidence for a significant association between subjective and neuroendocrine stress-reactivity in experimental setting and alcohol use and alcohol craving in everyday-life settings during a one-year ambulatory assessment phase.

In line with previous studies [17, 39], we observed significant effects of psychosocial stress on subjective and neuroendocrine stress responses, as well as on alcohol craving with higher values in the psychosocial stress group versus the physical stress and control group. With regards to the magnitude of the observed effects, psychosocial stress, in combination with alcohol cue-exposure, had the strongest effect on neuroendocrine cortisol reactivity, followed by subjective stress and craving. In addition, the effects of psychosocial stress on cortisol reactivity endured throughout the experimental session. This stood in contrast to the transient effects of psychosocial stress exposure on subjective stress responses and alcohol craving. Our observation can be explained by the previously observed properties of the cortisol response, which showed a delay of several minutes until onset and continued elevation after discontinuation of a stressor [8, 40]. Previous studies repeatedly reported altered cortisol levels in individuals with AUD [17]. This effect however seems to be dependent on the severity of AUD. Similarly, in our sample with a majority of mild to moderate AUD participants (81%) we found an association of increased stress- and cue-induced cortisol levels and the amount of consumed alcohol while findings with samples in a later stage of AUD reported that relapsers showed a blunted cortisol response to psychosocial stress [1, 13, 41]. Additionally, a more recent study reported that higher adrenal sensitivity (i.e. the cortisol to corticotropin ratio) predicted time to relapse [42].

Current results are in line with findings by Kwako et al. [17], who investigated the effects of a sequential stress and alcohol cue-exposure versus guided imagery of stressful and neutral conditions on alcohol craving, subjective and neuroendocrine stress responses in comorbid alcohol dependence and post-traumatic stress disorder. They found that both, the TSST combined with cue-exposure and the imagery of stressful situations induced craving for alcohol, subjective distress and anxiety. However, only the TSST induced increases in cortisol levels, indicating that cortisol responses might be specific to the psychosocial evaluative aspects of the TSST. In addition to previous work, presented data also indicate that psychosocial stress, induced by the TSST, yields different neuroendocrine stress responses when compared to a physical stressor (i.e. endurance exercise bike training), which induces similar physiological arousal. The investigation of a physical stressor, which was modeled to correspond to everyday-life physical stressors might also provide additional insight in the distinct effects of various forms of stress in AUD and provide increased external validity, compared to other experimental stress models (e.g. cold pressor test).

In accordance with previous studies, we observed a significant association between individual stress-reactivity and alcohol consumption during the follow-up [42]. Previous studies reported that individuals with severe AUD, who demonstrated the highest cortisol responsivity to corticotropin (considered a proxy for “adrenal sensitivity”) during a neutral, relaxing condition showed the shortest time to alcohol relapse [42]. Badrick et al. [43] analyzed the association between alcohol consumption and HPA axis activity and found in a large sample that increased alcohol consumption was predicted by higher cortisol levels. Recent studies that investigated the effect of a real-life stressor, the COVID19 pandemic, on cortisol reactivity and alcohol consumption replicated these findings [44]. In this study, higher cortisol levels predicted higher motivation to drink alcohol. Further research showed that the exposure to stress and alcohol cues predicted increased following alcohol intake in heavy drinkers compared to moderate drinkers [13, 45–47]. These studies confirmed the link between higher cortisol levels and higher alcohol consumption in individuals with different consumption patterns (recreational, binge/ heavy and chronic users), highlighting the interaction between stress reactivity and alcohol use. The current study adds to this evidence by demonstrating an association between neuroendocrine cortisol reactivity and alcohol consumption in individuals with light to severe AUD in everyday life settings during a one-year ambulatory assessment phase. We did not observe significant associations between either subjective stress reactivity or alcohol craving with alcohol consumption during the ambulatory phase. Instead, we found a significant association between subjective stress responses and alcohol craving during follow-up, analyzing associations between cortisol, subjective stress and alcohol craving in everyday life settings during a one-year ambulatory assessment phase. The distinct associations observed between subjective and neuroendocrine stress-reactivity and alcohol use and alcohol craving during the ambulatory phase are in line with previous studies that reported a disalignment of the subjective stress response and the neuroendocrine cortisol response in healthy individuals and individuals with AUD [14, 45, 48–50]. Our findings indicate that the impact of stress on AUD occurs via two distinct pathways – a subjective and a neuroendocrine pathway. This notion is in accordance with previous studies identifying distinct pathways of stress-reactivity [49, 51] and the current observation of a dissociation between subjective and neuroendocrine stress effects. Hellhammer et al. (2009) already proposed different pathways of stress-reactivity, which was supported by following studies that demonstrated a divergence and distinct pattern of parameters capturing subjective and neuroendocrine stress responses [11, 17, 49, 52]. In line with current findings, Clay & Parker [53] reported that physiological stress markers, including cortisol, predicted alcohol consumption, while psychological stress responses and alcohol craving did not. They speculated that stress-induced alcohol craving is predictive of alcohol use only in later stages of the addiction cycle, in accordance with the withdrawal state of addiction proposed by Koob and Volkow [54]. Our findings additionally suggest that two pathways of stress-reactivity differentially contribute to alcohol craving and alcohol use in earlier stages of AUD with the majority of our sample (81%) meeting criteria of a light to moderate AUD. Recent findings [55] demonstrated that the exposure to a stressful event on a particular day predicted increased craving on that day and that such increases in craving predicted the likelihood of drinking the next day in individuals with AUD. In contrast, Thomas et al. [56] reported that exposure to alcohol cues increased subjective craving in a sample of non-treatment-seeking individuals with severe AUD, while a preceding psychosocial stressor did not. These findings suggest that neuroendocrine and subjective stress pathways might be subject to dynamic adaptations in the course of AUD.

Taken together, presented findings indicate that two distinct stress-related pathways differently contribute to alcohol craving and alcohol use in individuals with mostly mild to moderate AUD. From a clinical perspective, individuals that consciously experience subjective stress and following craving as antecedents of alcohol use might benefit from psychotherapeutic interventions that promote stress-management skills, mindfulness and craving coping skills and treatments that target stress- and cue-reactivity [57]. On the other hand, individuals with dysregulated neuroendocrine stress responses might benefit from therapeutic strategies that enhance their perception of associated triggers and pharmacological interventions that attenuate HPA axis reactivity or glucocorticoid actions (e.g. mifepristone or esitalopram) [58, 59], even though robust clinical data on the efficacy of such strategies is still scarce [60–62]. Furthermore, informing individuals with AUD about the association between stress pathways, craving and alcohol use and monitoring of the associations between the three – e.g. via electronic diaries – could facilitate the identification of and coping with high-risk drinking situations and associated triggers.

### Strengths and limitations

The strengths of the study are the randomized-controlled study design and the investigation of a well-characterized study sample of individuals with mainly mild to moderate AUD. The focus on this stage of AUD might limit the findings presented here such that no conclusions might be drawn on clinical populations including individuals with severe AUD. But when including AUD-criteria in our analysis the found effects were robust. By choosing a parallel design over a cross-over design we aimed to avoid carry-over and repetition effects and enhance feasibility and reduce participant burden. Even though a carry-over design might have provided higher power, the current study was sufficiently powered to detect meaningful (small to medium) effects of the intervention on the outcomes of interest. Previous studies indicated several additional sources of variability for individual cortisol responses. Importantly, strong diurnal influences on cortisol levels were reported [63]. Considering the onset times of the experimental procedure, childhood trauma and age as covariates in the statistical models did not change the significance of the results, supporting the robustness of the findings. In addition, gender effects on cortisol-reactivity have been repeatedly demonstrated in which women’s cortisol response to psychosocial stress was described as about 50-150% to baseline and men’s about 200-400% [64–66]. In our data we also saw an effect of age on cortisol but the main effect remained robust. Further, even though a decreasing EMA compliance rate over time was noted, the response rate was still high considering the time frame of one year.

## Conclusion

Current results provide further evidence for significant interactions between psychosocial stress and alcohol cue-exposure on neuroendocrine and subjective stress responses and alcohol craving in mild to severe AUD. In addition, our results highlight the differential contribution of neuroendocrine and subjective stress response systems and their reactivity to the occurrence of alcohol consumption and alcohol craving in real-life settings. Furthermore, our results support previous theoretical frameworks proposing distinct neuroendocrine and subjective stress response systems. Current results indicate that individuals with AUD might benefit from interventions targeting neuroendocrine and subjective stress reactivity.

## Supplementary information

Supplemental material

## Data Availability

Data will be made available upon request.
