# Supplementary material for: Individual stress reactivity predicts alcohol craving and alcohol consumption in alcohol use disorder in experimental and real-life settings
Source: Transl Psychiatry. 2025 Jul 3;15:226. doi: 10.1038/s41398-025-03447-8 (PMC12229504; doi:10.1038/s41398-025-03447-8)
Supplement: Supplementary file 1 — Supplemental material [file 41398_2025_3447_MOESM1_ESM.docx]

**Supplements**

*Individual stress reactivity predicts alcohol craving and alcohol consumption in alcohol use disorder in experimental and real-life settings*

**Content:**

- **Supplementary Methods**
  - Eligibility criteria
- Experimental procedure
- Cortisol pre-analysis
- EMA assessments
- **Supplementary Tables**
  - - **Supplementary Table S1.** Sample characteristics: Comparison of the current sub-sample to the remaining sample
    - **Supplementary Table S2.** Multilevel Modelling Results for the effect of experimental intervention on blood pressure
    - **Supplementary Table S3.** Multilevel Modelling Results for cortisol prediction, including starting time of the experiment
    - **Supplementary Table S4.** Multilevel Modelling Results for identifying associations to real-life alcohol craving and consumption
- **Supplementary Figures**
  - **Supplementary Figure S1.** Depiction of main results (craving)
  - **Supplementary Figure S2.** Depiction of main results (alcohol consumption)
  - **Supplementary Figure S3.** CONSORT study flow chart
- **References**

**Supplementary Methods**

**Addition: Eligibility criteria**

Further eligibility criteria included: i) age between 16 to 65 years, ii) the ability to understand the study protocol, and to give written informed consent. Exclusion criteria included i) the use of medication interacting with the central nervous system in the last ten days or if the use dates back less than five half-life periods, ii) contraindications for undergoing fMRI, iii) diagnosis of bipolar disorder, psychotic disorder, schizophrenia or disorder from the schizophrenic spectrum, or the current use of drugs (except recreational cannabis use) or a diagnosis of substance use disorder (except for alcohol, nicotine, and cannabis) according to the DSM-5, iv) history of severe head trauma, v) history of central nervous system diseases (e.g. epilepsy, dementia, Parkinson’s disease, multiple sclerosis), vi) pregnancy or breastfeeding.

**Addition: Experimental procedure**

Start time of the assessments varied between 9am and 4pm but this was controlled for in the analysis. Participants were asked to abstain from alcohol since the previous evening, from food for two hours and from drinks for 30 minutes before the start of the study assessment. At the beginning of the visit, a urine drug test and an alcohol breathing test were performed to ensure abstinence. Furthermore, participants completed a series of questionnaires, including the Clinical Institute Withdrawal Assessment for Alcohol (CIWA) to ensure the absence of acute withdrawal symptoms, the Fagerström Test for Nicotine Dependence (FTND) or the e-cigarette Fagerström Test of Cigarette Dependence (e-FTCD), depending on the type of cigarettes they smoke, the Alcohol Dependence Scale (ADS) and the Obsessive Compulsive Drinking Scale (OCDS-G). Participants then drank 200ml grape juice (including approx. 9-16g sugar per 100 ml) to standardize responsiveness of HPA axis (1,2) for subsequent cortisol assessments and were allowed to rest for 30-minutes, in order to yield a baseline arousal level.

**Cortisol pre-analysis**

Saliva samples were frozen and stored at -20 °C until analysis. After thawing, salivettes were centrifuged at 3,000 rpm for 5 min, which resulted in a clear supernatant of low viscosity. Salivary concentrations were measured using commercially available chemiluminescence immunoassay with high sensitivity (Tecan - IBL International, Hamburg, Germany; catalogue number R62111). The intra and interassay coefficients for cortisol were below 1,0% and 2,0%, respectively.

**EMA assessment**

Items: Craving request was prompted every second day on the smartphone with the question “How much alcohol craving (desire for alcohol in times when you did not consume alcohol) did you experience in the last 24 hours on average?”. Responding options were given on a 7 point Likert scale from 1 = none to 7 = extreme craving.

Alcohol consumption request was prompted every second day on the smartphone with the questions i) “Think about yesterday. Which and how many alcoholic drinks did you have?” (Drop-down list of alcoholic drinks with size and volume-% specifications + request for amount of checked drinks) and ii)“Think about the day before yesterday. Which and how many alcoholic drinks did you have?” (Drop-down list of alcoholic drinks with size and volume-% specifications + request for amount of checked drinks).

Accuracy of self-reported drinking: Participants did not receive an incentive for answering the prompts, data were collected pseudonymized and the collection of the data was purely observational i.e. participants did not receive a feedback from a clinician.

**Supplementary Tables**

Supplementary Table S1. Demographic data, alcohol use, and severity measures for participants of the general cohort study of the collaborative research consortium without the participants of the current study (N=155) and for participants enrolled in the current sub-study (N=121).

| **Sample** | **Cohort**  **sample** | **Study**  **sample** |  |  |
| --- | --- | --- | --- | --- |
|  | **(n=155)** | **(n=121)** | **Statistics** | **Significance** |
| *Demographical variables* |  |  |  |  |
| Gender (female; male) | 53; 102 | 47; 74 | Z = -0.80 | p = .451 |
| Age (years) | 39.29 (13.98) | 38.41 (13.21) | T(274) = -0.54 | p = .593 |
| *Substance use patterns* |  |  |  |  |
| AUD criteria last 12 months | 3.99 (1.67) | 4.01 (1.55) | *T*(274) = 0.12 | *p* = .914 |
| AUDIT | 15.49 (5.36) | 15.36 (5.58) | *T*(240) *= -*0.19 | *p* = .853 |
| Alcohol consumption - last 3 months [g alcohol/day] | 6.06 (4.12) | 6.15 (3.43) | *T*(274) *=* 0.19 | *p* = .850 |
| Alcohol consumption - typical weekday [g alcohol/day] | 3.42 (2.75) | 3.57 (2.56) | *T*(274) *=* 0.46 | *p* = .649 |
| Alcohol consumption - typical weekend [g alcohol/day] | 7.93 (4.86) | 7.32 (3.98) | *T*(274) *=* -1.13 | *p* = .260 |
| FTND | 2.36 (2.34) | 1.66 (2.15) | *T(*77) = -1.38 | *p* = .172 |
| *Clinical scales* |  |  |  |  |
| PSS | 16.22 (7.40) | 16.11 (7.12) | *T* (236) = -0.12 | p = .906 |
| BSI | 21.25 (20.60) | 21.27 (18.13) | *T* (237) = 0.01 | p = .995 |
| STAI Trait | 39.88 (11.08) | 39.02 (10.77) | T(253) = -0.63 | p = .529 |
| CTS | 7.68 (2.91) | 7.70 (3.26) | *T*(274) = 0.05 | *p* = .960 |

AUDIT = Alcohol Use Disorders Identification Test; FTND = Fagerstroem Test for Nicotine Dependence; Scale; BSI = Brief Symptom Inventory; STAI = State-Trait-Anxiety Inventory; CTS = Childhood Trauma Screener;

Note: Gender: None of the participants assigned themselves to “divers”

SD = standard deviation; * = significant differences p < 0.05; ° = significant post-hoc test p < 0.05

| **Supplementary Table S2.** Multilevel Modelling Results for the effect of experimental intervention on blood pressure (systolic). | | | | |
| --- | --- | --- | --- | --- |
|  | F (df1, df2) | p |  |  |
| **Blood pressure (systolic)** |  |  |  |  |
| Intercept | 7149.31 (1, 116.35) | **< .001** |  |  |
| Group | 0.45 (2, 116.35) | .639 |  |  |
| Timepoint (T) | 29.78 (4, 462.51) | **< .001** |  |  |
| Group * T | 2.42 (8, 462.5) | **.014** |  |  |
| Group = stress condition; Repeated assessments: blood pressure (systolic) n=5;  95% CI, 95% confidence interval. p < .05 in bold | | | | |

| **Supplementary Table S3.** Multilevel Modelling Results for cortisol prediction, including starting time of the experiment. | | | | | |
| --- | --- | --- | --- | --- | --- |
|  | β [95% CI] | p | F (df1, df2) | p |  |
| **Cortisol (log)** |  |  |  |  |  |
| Intercept | 2.11 [1.39;2.82] | **<.001** | 42.66 (1;128.18) | **<.001** |  |
| Gender |  |  | 9.06 (1;93.65) | **.003** |  |
| Male | 0.28 [0.1;0.46] | **.003** |  |  |  |
| Female | [Reference] |  |  |  |  |
| Group |  |  | 3.46 (2;90.82) | **.036** |  |
| TSST | 0.28 [0.06;0.49] | **.012** |  |  |  |
| TEEB | 0.07 [-0.14;0.29] | .500 |  |  |  |
| CTRL | [Reference] |  |  |  |  |
| Age | -0.01 [-0.01;0] |  | 3.61 (1;99.19) | .060 |  |
| AUD criteria | 0.07 [0.01;0.13] |  | 5.72 (1;91.2) | **.019** |  |
| PSS | 0.01 [-0.01;0.02] |  | 0.97 (1;91.1) | .327 |  |
| Timepoint (T) | 0.04 [-0,35;0.43] |  | 1.61 (2;220.44) | .203 |  |
| T1 | [Reference] |  |  |  |  |
| T3 | 0.04 [-0.06;0.14] | .474 |  |  |  |
| T4 | 0.09 [-0.01;0.19] | .078 |  |  |  |
| AUQ | -0.01 [-0.01;0] |  | 1.97 (1;289.95) | .161 |  |
| PASA | 0.01 [-0.07;0.1] |  | 0.1 (1;95.18) | .755 |  |
| BP (sys) | 0. [0;0] |  | 0.04 (1;254.48) | .835 |  |
| Starting time | -2,482e^-5^ [-3.489e^-5^;-1.475e^-5^] |  | 23.98 (1;90.82) | **<.001** |  |
| Group = stress condition; TSST = Trier Social Stress Test; TEEB = Training on an Endurance Exercise Bike; CTRL = Control group; AUD = alcohol use disorder; PSS = perceived stress scale; AUQ = alcohol urge questionnaire; PASA = primary appraisal secondary appraisal; BP (sys) = systolic blood pressure; Starting time = Starting time of the experimental procedure;  95% CI, 95% confidence interval. p < .05 in bold | | | | | |

| **Supplementary Table S4.** Multilevel Modeling Results for identifying associations to real-life alcohol craving and alcohol consumption (EMA period: One year; craving i= 11257, alcohol consumption: i=22653). | | | | | |
| --- | --- | --- | --- | --- | --- |
|  | β [95% CI] | p | F (df1, df2) | p |  |
| **Craving** |  |  |  |  |  |
| Intercept | 0.21 [-1.35;1.76] | .794 | 0.03 (1;94.51) | .867 |  |
| Gender |  |  | 1.32 (1;95.04) | .253 |  |
| Male | -0.2 [-0.55;0.15] |  |  |  |  |
| Female | [Reference] |  |  |  |  |
| Group |  |  | 5.85 (2;94.7) | **.004** |  |
| TSST | -0.65 [-1.03;-0.27] | **.001** |  |  |  |
| TEEB | -0.21 [-0.6;0.18] | .280 |  |  |  |
| CTRL | [Reference] |  |  |  |  |
| Age | 0.02 [0;0.03] |  | 6.27 (1;94.37) | **.014** |  |
| Weekend days versus weekdays |  |  | 33.86 (1;10487.99) | **<.001** |  |
| Weekend days | 0.1 [0.07;0.14] |  |  |  |  |
| Weekdays | [Reference] |  |  |  |  |
| AUD criteria | 0.05 [-0.06;0.15] |  | 0.73 (1;94.47) | .396 |  |
| PSS | 0.02 [-0.01;0.04] |  | 1.81 (1;94.07) | .181 |  |
| Cortisol (log) | 0.07 [-0.27;0.42] |  | 0.19 (1;95.42) | .666 |  |
| PASA | 0.16 [0.01;0.32] |  | 4.39 (1;94.41) | .**039** |  |
| AUQ | 0.05 [0.02;0.07] |  | 13.75 (1;95.68) | **<.001** |  |
| BP (sys) | 0 [-0.01;0.02] |  | 0.82 (1;93.38) | .367 |  |
|  |  |  |  |  |  |
| **Alcohol consumption** |  |  |  |  |  |
| Intercept | -53.18 [-95.99;-10.37] | .015 | 4.76 (1;95.09) | **.032** |  |
| Gender |  |  | 0.41 (1;95.72) | .525 |  |
| Male | 3.1 [-6.56;12.77] |  |  |  |  |
| Female | [Reference] |  |  |  |  |
| Group |  |  | 0.39 (2;95.32) | .678 |  |
| TSST | -2.73 [-13.22;7.76] | .607 |  |  |  |
| TEEB | -4.72 [-15.44;6] | .384 |  |  |  |
| CTRL | [Reference] |  |  |  |  |
| Age  Weekend days versus weekdays | 0.39 [0.02;0.77] |  | 4.42 (1;94.91)  680.56 (1;21101.21) | **.038**  **<.001** |  |
| Weekend days | 14.03 [12.98;15.09] |  |  |  |  |
| Weekdays | [Reference] |  |  |  |  |
| AUD criteria | -1.2 [-4.18;1.78] |  | 0.64 (1;95.06) | .426 |  |
| PSS | 0.6 [-0.13;1.33] |  | 2.64 (1;94.57) | .107 |  |
| Cortisol (log) | 9.76 [0.3;19.23] |  | 4.19 (1;96.15) | **.043** |  |
| PASA | -2.24 [-6.51;2.03] |  | 1.09 (1;94.97) | .300 |  |
| AUQ | 0.12 [-0.57;0.81] |  | 0.12 (1;96.41) | .733 |  |
| BP (sys) | 0.32 [0.04;0.61] |  | 5.03 (1;93.77) | **.027** |  |
| AUD = alcohol use disorder; AUQ = alcohol urge questionnaire; BP (sys) = systolic blood pressure; group = stress condition; PASA = primary appraisal secondary appraisal; PSS = perceived stress scale;  Weekend days = Friday to Sunday;  95% CI, 95% confidence interval. p < .05 in bold | | | | | |

**Supplementary Figures**

**Supplementary Figure S1.** Depiction of the association of *craving* (estimated marginal means) with PASA and Cortisol (mean, +/- STD).


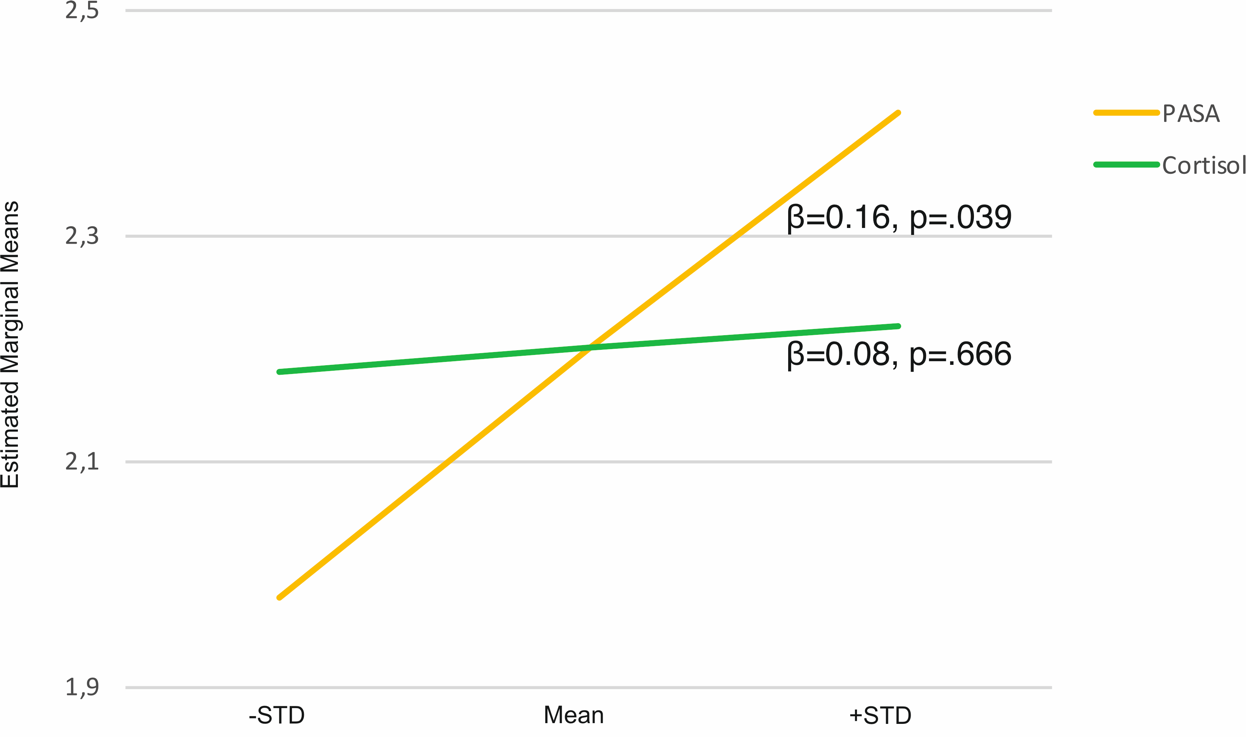


**Supplementary Figure S2.** Depiction of the association of *alcohol consumption* (estimated marginal means) with PASA and Cortisol (mean, +/- STD).


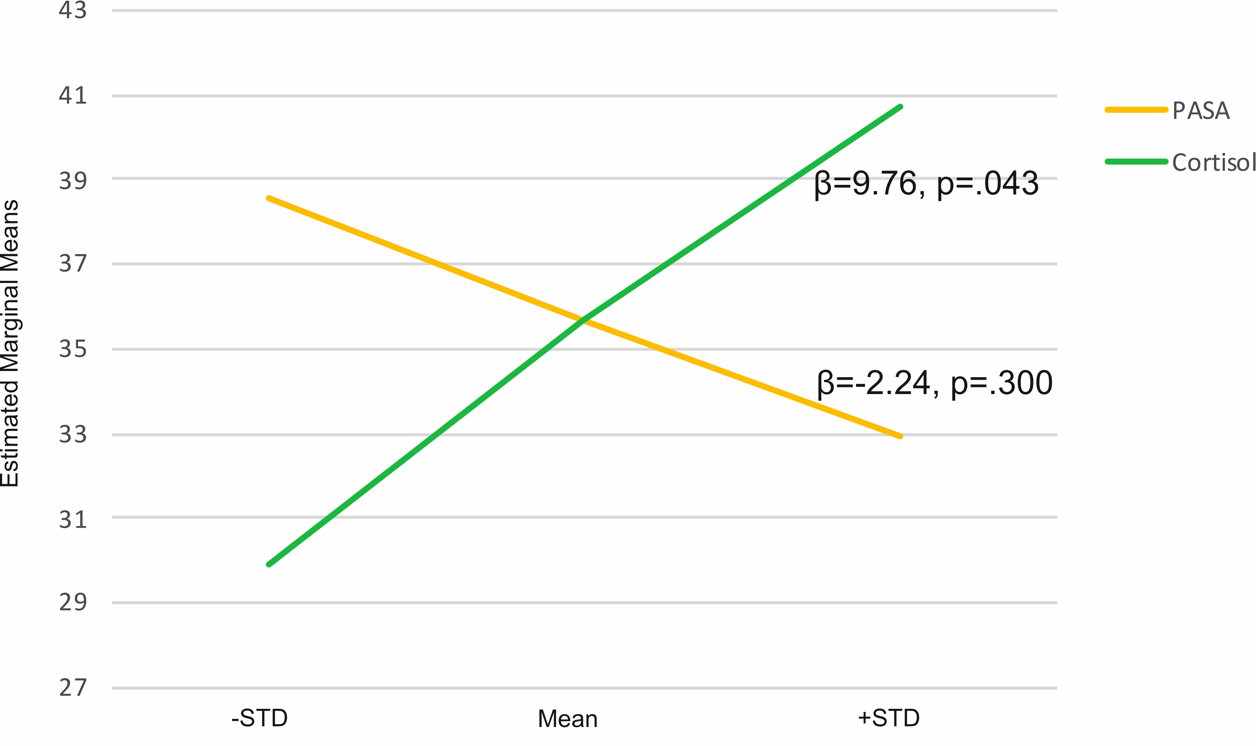


**Supplementary Figure S3.** CONSORT study flow chart


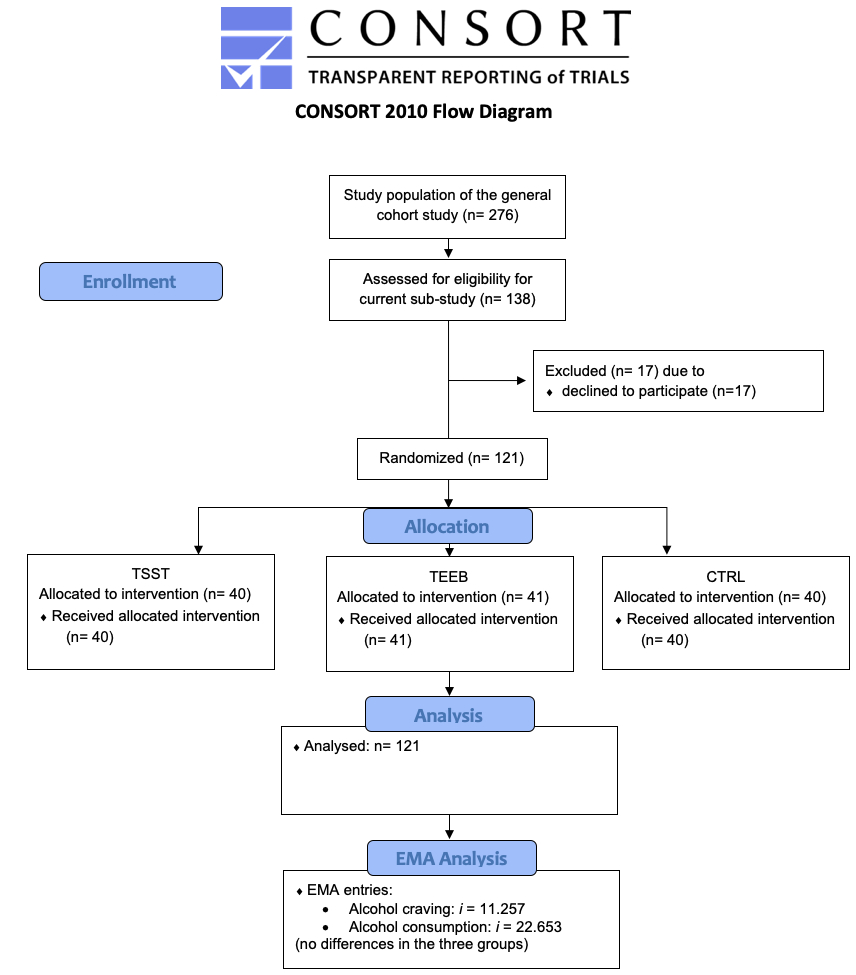


**References**

1. Kirschbaum C, Bono EG, Rohleder N, Gessner C, Pirke KM, Salvador A, Hellhammer DH (1997): Effects of Fasting and Glucose Load on Free Cortisol Responses to Stress and Nicotine. 82: 5.

2. Kudielka BM, Hellhammer DH, Wüst S (2009): Why do we respond so differently? Reviewing determinants of human salivary cortisol responses to challenge. *Psychoneuroendocrinology* 34: 2–18.
